# Transcriptome Analysis of Choke Stroma and Asymptomatic Inflorescence Tissues Reveals Changes in Gene Expression in Both *Epichloë* *festucae* and Its Host Plant *Festuca rubra* subsp. *rubra*

**DOI:** 10.3390/microorganisms7110567

**Published:** 2019-11-16

**Authors:** Ruying Wang, Bruce B. Clarke, Faith C. Belanger

**Affiliations:** Department of Plant Biology, Rutgers University, New Brunswick, NJ 08901, USA; ruying.wang@rutgers.edu (R.W.); bruce.clarke@rutgers.edu (B.B.C.)

**Keywords:** endophyte, strong creeping red fescue, symbiosis

## Abstract

Many cool-season grasses have symbiotic relationships with *Epichloë* (Ascomycota, Clavicipitaceae) fungal endophytes that inhabit the intercellular spaces of the above-ground parts of the host plants. The presence of the *Epichloë* endophytes is generally beneficial to the hosts due to enhanced tolerance to biotic and abiotic stresses conferred by the endophytes. Many *Epichloë* spp. are asexual, and those infections always remain asymptomatic. However, some *Epichloë* spp. have a sexual stage and produce a macroscopic fruiting body, a stroma, that envelops the developing inflorescence causing a syndrome termed “choke disease”. Here, we report a fungal and plant gene expression analysis of choke stroma tissue and asymptomatic inflorescence tissue of *Epichloë* *festucae*-infected strong creeping red fescue (*Festuca rubra* subsp. *rubra*). Hundreds of fungal genes and over 10% of the plant genes were differentially expressed when comparing the two tissue types. The differentially expressed fungal genes in the choke stroma tissue indicated a change in carbohydrate and lipid metabolism, as well as a change in expression of numerous genes for candidate effector proteins. Plant stress-related genes were up-regulated in the stroma tissue, suggesting the plant host was responding to the epiphytic stage of *E. festucae* as a pathogen.

## 1. Introduction

Many cool-season grasses have symbiotic relationships with *Epichloë* (Ascomycota, Clavicipitaceae) fungal endophytes, which inhabit the intercellular spaces of the above-ground parts of the host plants (see reviews [1,2]). The presence of the fungal endophytes is generally beneficial to the hosts due to the enhanced tolerance to biotic and abiotic stresses conferred by the endophytes (see reviews [3,4]). Endophyte-infected forage and turf grasses are marketed for their insect deterrence and toxicity to pests or non-toxicity to livestock [5].

**Figure 1:**
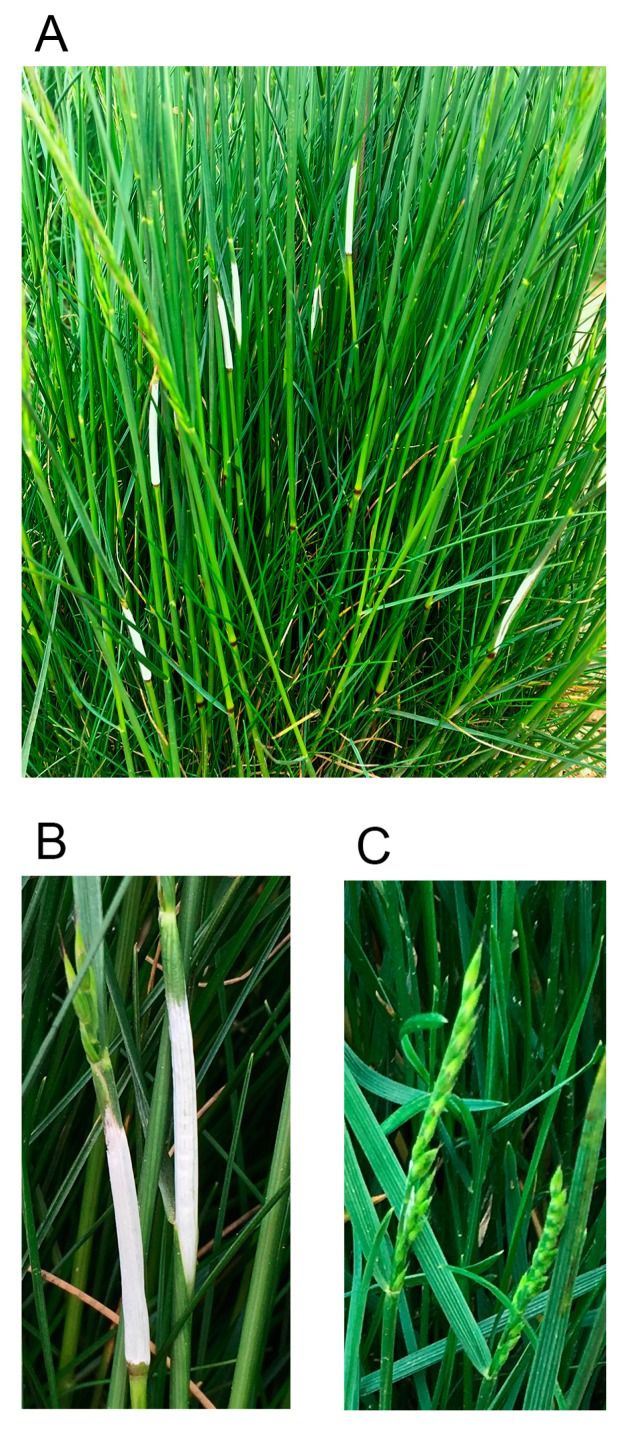
(**A**) Strong creeping red fescue plant with choke stromata and asymptomatic inflorescences. (**B**) Choke stromata. (**C**) Asymptomatic inflorescences.

Leuchtmann et al. presented a comprehensive description of 43 *Epichloë* taxa [6], and new taxa are frequently discovered [7]. Most of the known *Epichloë* spp. are asexual hybrid species. Plants infected with the asexual hybrid *Epichloë* spp. are generally symptomless throughout their lifecycle, and the endophytes are dispersed to the next plant generation in seeds by infecting the ovary and developing embryo [8]. In contrast, most of the haploid *Epichloë* spp. have a sexual cycle, which is strictly linked to flowering of the host plant. During development of the flowering tiller, the normally asymptomatic intercellular fungal mycelium proliferates extensively and eventually forms an external stroma, the fungal fruiting structure, which envelops the developing inflorescence (Figure 1). Because the development of the inflorescence is physically constrained by the fungus, this syndrome is referred to as “choke disease”. Tadych et al. [9] have presented a comprehensive review of the life cycle of the stroma-forming *Epichloë* endophytes. The *Epichloë* sexual cycle occurs when heterothallic conidia, which function as gametes, are transferred between stromata, often carried by *Botanophila* spp. flies. After fertilization, perithecia develop containing the ascospores, which may function to infect new grass hosts.

Choke disease can be a serious problem on commercially important grasses, since it reduces seed yields. The turf grasses Chewings fescue (*Festuca rubra* subsp. *commutata*) and strong creeping red fescue (*F. rubra* subsp. *rubra*), and the forage orchardgrass (*Dactylis glomerata*) can experience choke disease. In Oregon, losses due to choke disease in orchardgrass in 2004 were estimated to be 0.8 million dollars [10]. The use of fungicides to control choke disease in orchardgrass caused by *Epichloë typhina* was ineffective [11]. In the fine fescues, years of selective breeding for endophyte-infected plants not exhibiting choke symptoms have successfully reduced the incidence of choke disease in new cultivars, but choke disease can still be a problem [12]. White classified the *Epichloë*/grass host symbiosis into three types [13]. In type 1 associations, such as that in orchardgrass, stromata are produced on all endophyte-infected inflorescences, and therefore, there is no seed transmission of the endophyte. In type 2 associations, such as that in *F. rubra*, stromata are produced on only some of the inflorescences, and the endophyte is transmitted through seed produced on non-choked inflorescences. In type 3 associations, such as those with the hybrid *Epichloë* spp., stromata are never formed.

What triggers the development of a fungal stroma in response to the host flowering is not known. The change from asymptomatic endophytic to macroscopic epiphytic mycelium must involve an increase in fungal growth rate, which suggests an increase in nutrient supply to the fungus. Kirby proposed that the developmental stage and growth rate of the host’s flowering apex were critical factors in stroma development [14]. He reported that the rapid growth of the fungus began after the floral apex developed to the double-ridge stage. If the growth rate of the floral apex was slow, then the fungus would be in contact with the relatively large volume of plant meristematic cells long enough to absorb adequate nutrients for enhanced fungal growth. Field research indicated that nitrogen fertilization at rates used commercially to maximize seed yield could reduce choke disease in *E. festucae*-infected Chewings fescue [15]. These findings support the hypothesis that a faster growing floral apex could escape stroma development. However, there has not been a subsequent exploration of this hypothesis. A complementary hypothesis presented by White et al. was that stroma-forming *Epichloë* isolates are better able to utilize host sugars for rapid growth [16]. Lam et al. [17] reported dramatically elevated fungal invertase activity in stroma tissue, which would likely improve the ability of the fungus to access sucrose produced by the plant and would support rapid mycelial proliferation. Concomitant with the change in fungal growth rate in stromata is a change in growth habit, from restricted growth in the asymptomatic tissues [18,19] to unrestricted growth in stromata [20,21].

Bucheli and Leuchtmann reported isozyme data of *Epichloë sylvatica* stroma and non-stroma-forming isolates from *Brachypodium sylvaticum*, and concluded there was genetic differentiation between the isolates [22]. Meijer and Leuchtmann proposed that the fungal genome controls stroma formation and that the choked and non-choked tillers were infected with different strains of *E. sylvatica* in *B. sylvaticum* populations, where stromata develop only on some inflorescences of an individual plant [23]. Data from Chewings and strong creeping red fescues cross-inoculated with *Epichloë* isolates suggested incidence of choke disease may depend on both the host plant genotype and the *Epichloë* endophyte genotype [24]. Both of these possibilities fit the hypothesis that some signal or metabolite from the host that is present in the flowering stage may be involved and that the fungus must be able to respond to such a signal for stromata development to occur.

Stromata formation by *Epichloë* spp. is largely associated with host flowering, but there is a report of stromata formation on vegetative leaves of *E. typhina* infected perennial ryegrass [20] and on leaf sheaths of *Epichloë stromatolonga*-infected *Calamagrostis epigeios* [25]. Also, stromata formation is generally restricted to haploid *Epichloë* spp. However, there is a report of stromata formation by an interspecific hybrid between *E. typhina* and *E. festucae* var. *lolii* on perennial ryegrass inflorescences [21]. In these three cases, no perithecia or ascospores were formed. These exceptional cases raise additional questions about the signal or metabolic conditions that lead to initiation of stromata formation and requirements for production of the sexual structures. 

As a next step in investigating the mechanism underlying the development of choke disease, we carried out a transcriptome study to determine the differentially expressed plant and fungal genes between asymptomatic inflorescence tissue and choke stroma tissue in *E. festucae*-infected strong creeping red fescue. Our objectives were to determine which fungal genes may be underlying the increase in growth rate and how the plant tissue respond to that change.

## 2. Materials and Methods

### 2.1. Sample Preparation

Three biological replicates of healthy developing asymptomatic endophyte-infected inflorescences and choke stromata were collected from the same strong creeping red fescue plant (plant 6035-5 A10-484) from Rutgers research farm in Adephia, NJ on 10 May 2016. The choke stromata samples included both the epiphytic fungal tissue and the internal arrested developing inflorescence tissue. Healthy developing inflorescences were collected before anthesis, and choke stromata were collected before the development of perithecia, when the fungal stroma turns yellow to orange color. Samples were immediately frozen on dry ice and then stored at −80 °C before RNA extraction. Total RNA was extracted from both tissue types using ZR Fungal/Bacterial RNA MiniPrep (Zymo Research, Orange, CA, USA) and treated with DNase to remove DNA, following the manufacturer’s recommendations.

### 2.2. RNA Sequencing

RNA-Seq was performed by the Waksman Genomics Core Facility, Rutgers University. In brief, RNA concentration and integrity was verified using BioAnalyzer 2100 with RNA 6000 Nano Labchips, according to manufacturer’s instructions (Agilent Technologies, Palo Alto, CA, USA). Samples of RNA had 28S/18S ratios ranging from 1.8 to 2.0 and RNA Integrity Number values of 7.0 to 9.0. PolyA RNA was isolated from 5–10 μg total RNA with oligo(dT) beads using two rounds of oligo-dT purification. Fifty to 100 ng mRNA was used for Illumina library preparation.

Directional cDNA libraries were prepared using the dUTP method with a NEB Ultra directional RNA Library Prep for Illumina kit (New England BioLabs Inc., Ipswich, MA, USA). Each sample was ligated with different indexes and amplified with 12 PCR cycles. The quality and quantity of cDNA libraries were evaluated using Qubit 2.0 (Invitrogen, Life Technologies, Carlsbad, CA, USA), BioAnalyzer 2100 with the DNA 1000 kit, and real-time PCR using the KAPA Library Quantification Kit (Kapa Biosystems, Boston, MA, USA). Adapter-ligated cDNA fragment libraries were pooled together and loaded into an Illumina NextSeq500 using the NextSeq 500/550 High Output Kit version 3, according to the manufacturer’s protocol (Illumina, San Diego, CA, USA).

### 2.3. Data Analysis

Raw sequence data were de-multiplexed and FastQC software (v0.11.4) was applied for the quality control (QC) of raw sequences. Fastq output files were used for all downstream applications. Adaptor sequences and reads shorter than 36 bp and/or reads with a quality score lower than 15 were removed from the dataset using Cutadapt software (v1.3). Data were submitted to NCBI (BioProject PRJNA490735). The trimmed reads were then mapped to the mitochondrion and chloroplast sequences (GenBank accessions: NC_009950.1 and JX999996.1) of *L. perenne*―the closest species to *F. rubra* subsp. *rubra* for which there are mitochondrion and chloroplast sequences publicly available. The mapped reads were discarded and all the unmapped reads were retained for further analysis.

The filtered reads were first mapped to the reference genome *E*. *festucae* E2368 from the *Epichloë festucae* Genome Project [26]. The mapping was done with CLC Genomics Workbench v10.1.1 with the minimum read length fraction and the read similarity fraction both set to 0.9. Reads that mapped to more than one contig, which were less than 0.05% of the mapped reads, were not counted. Differential expression was done using the RNA-Seq Analysis tool with across group comparisons (ANOVA-like). Genes with normalized reads averaged across replications less than 10 RPKM in both symptomatic and asymptomatic tissues were not used for differential expression analysis. Significance thresholds were set to false discovery rate (FDR)-adjusted *p*-value lower than 0.01 and fold change greater than 4 (Log_2_ fold change of 2) difference in expression level. 

The remaining putative plant RNA-seq reads were pooled for assembling the strong creeping red fescue transcriptome using Trinity [27] and CLC Genomics Workbench. CD-HIT [28,29] with a threshold of 90% identity was used to reduce redundancy. The non-redundant assembly contigs were used as query for a homology search using BlastX against the NCBI non-redundant protein database (NR), applying a 10^−5^ e-value cutoff. Among the BlastX matches, 78 were to non-Viridiplantae species and these sequences were omitted from the assembly. 

The next step was to identify differentially expressed plant genes. For that, we separately mapped each library to the transcriptome assembly and counted the number of reads mapped to each contig. Differentially expressed genes were analyzed using the R Bioconductor package DESeq2 [30]. A table with the unique read counts per contig per library was used as input to DESeq2 and genes with an FDR-adjusted *p*-value lower than 0.01 were considered differentially expressed. 

Gene ontology (GO) terms were assigned to plant and fungal transcripts in Blast2GO (BioBam, Valencia, Spain). Plant GO slim categorization was generated for plant transcripts. Over-represented GO terms among the differentially expressed plant genes were identified by using Fisher’s Exact Test in Blast2GO with FDR < 0.05. No over-represented GO terms were identified among the differentially expressed fungal transcripts. 

## 3. Results and Discussion

### 3.1. Summary of RNA-Seq Data

The characteristics of the strong creeping red fescue transcriptome assembly are presented in Table 1. The fungal and plant mapping data are presented in Table 2. In the asymptomatic inflorescence tissue, the fungal reads were a small percentage, 1–1.5%, of the total reads. Similarly low levels of fungal transcripts in asymptomatic plant tissues, 0.01–3.41%, have been reported previously [31,32,33,34]. In contrast the fungal reads were a major percentage of the total reads, 76–83%, in the choke stroma samples, reflecting the increased fungal biomass in the stroma samples.

### 3.2. Epichloë festucae Gene Expression In Asymptomatic Inflorescence Tissue and Choke Stroma Tissue

There are 9324 annotated genes in the *E. festucae* 2368 genome [26]. Here, sequence reads were mapped to 9142 of the annotated genes. The 25 most abundantly expressed fungal genes in the asymptomatic inflorescence tissues and the choke stoma tissues are presented in Table 3 and Table 4, respectively. The gene expression profiles of the most abundantly expressed genes were dramatically altered when *E. festucae* switched from restricted, intercellular growth in the asymptomatic inflorescence tissue to the proliferative growth of the fungal stroma, which arrested growth of the host reproductive tissue. Only six genes were among the 25 most highly expressed genes in both tissue types—clock-controlled protein (EfM3.019720), translation elongation factor EF-1 alpha (EfM3.021690), Grg1 (EfM3.025530), a secreted serine/threonine-rich protein (EfM3.028690), polyubiquitin (EfM3.043630), and an uncharacterized protein (EfM3.072910). 

For both tissue types, most of the 25 most highly expressed fungal genes were differentially expressed—21 for the asymptomatic inflorescence tissue and 14 for the choke stroma tissue. In the asymptomatic inflorescence tissue, many of the abundantly expressed genes encode proteins of primary cellular metabolism, such as ubiquitin, ribosomal proteins, translation elongation factor EF-1 alpha, and glyceraldehyde 3-phosphate dehydrogenase. Surprisingly, most of these metabolic genes were differentially expressed, having significantly higher expression levels in the asymptomatic inflorescence tissue than in the choke stroma tissue.

Another category of highly expressed genes in the asymptomatic inflorescence tissue was that of candidate effector genes [35] (discussed more below), with specific candidate effector genes more highly expressed in the asymptomatic tissue than in the choke stroma tissue. One of the highly expressed candidate effector genes has also been functionally characterized as an antifungal protein gene, *Efe-afpA* (EfM3.063660) [39]. The purified *Efe*-AfpA protein has been shown to have activity against the dollar spot pathogen *Clarireedia jacksonii* [39] (formerly *Sclerotinia homoeocarpa* [45]).

In addition to the antifungal protein gene, other abundantly expressed genes in the asymptomatic inflorescence tissue have previously been reported in *E. festucae*. Nine of the abundantly expressed genes in the asymptomatic inflorescence tissue were reported as among the top 20 fungal genes in the *E. festucae*/strong creeping red fescue interaction in asymptomatic leaf sheath tissue [31]. The abundant *Epichloë*-specific genes NC12 (EfM3.018170) and *gigA* (NC25) (EfM3.028480) have been reported as abundantly expressed in leaf sheaths and blades of many *Epichloë* spp./grass associations [31,32,34,36,46]. The secreted hydrolytic enzymes chitinase and beta-1,6-glucanase have previously been characterized [38,40,41]. All of these genes were more highly expressed in the asymptomatic inflorescence tissue than in the choke stroma tissue. The *Epichloë* beta-1,6-glucanase gene was shown to be horizontally transferred to a cool season grass genome approximately 9–13 million years ago, and is now found in grass species from the sub-tribes Lolinae and Dactylidinae [47]. The plant beta-1,6-glucanase gene was found to be expressed in perennial ryegrass in all tissues examined, but was most highly expressed in root and flower tissues [47]. The evolutionary maintenance of an expressed horizontally transferred gene suggests it may have a role in the symbiotic association [47]. An *F. rubra* beta-1,6-glucanase gene has not been reported, but in the future, it would be interesting to examine the relative contributions of the fungal and plant gene expression in the asymptomatic inflorescence tissues and the choke stroma tissues.

Only three of the 25 most highly expressed genes in the choke stroma tissue have been characterized in *Epichloë*—*rhgA* (EfM3.030930), *esdC* (EfM3.019650), and a CuZn-superoxide dismutase (EfM3.075710) [42,43,44], none of which was differentially expressed. The functions of most of the other highly expressed genes in the choke stroma tissue are currently unknown. Five are candidate effector proteins [35] (discussed more below), distinct from those more highly expressed in the asymptomatic inflorescence tissue. Such a shift in expression of candidate effector proteins may be of significance to the shift in growth between asymptomatic to macroscopic that occurs in the development of the stroma tissue.

Another highly expressed gene that may relate to development of the stroma is aquaglyceroporin (EfM3.025350), which was more highly expressed in the stroma tissue than the asymptomatic inflorescence tissue. The annotation of the gene as “aquaglyceroporin” is based on the detection of a conserved domain through a Blast search at NCBI. In the *E. festucae* 2368 isolate genome sequence, the gene is annotated both as “aquaglyceroporin” and “aquaporin” [26] (http://csbio-l.csr.uky.edu/ef2011/). Fungal “aquaglyceroporins” often actually function as water channels [48], so functional characterization of the *E. festucae* gene would be required to determine the type of molecule transported. However, this is the only gene in the genome with the annotation of aquaporin or major intrinsic protein (http://csbio-l.csr.uky.edu/ef2011/), so it may function in water transport. There are several instances of fungal aquaglyceroporins functioning in water transport and their genes being up-regulated due to the increased demand for water in rapidly growing fruiting bodies [48]. Similarly, water would be essential for stroma development. White et al. reported that transpiration was enhanced in stromal leaves compared to non-stromal leaves, and would function to draw water through the stroma [49].

In addition to the abundant differentially expressed genes discussed above, full lists of the differentially expressed genes are presented in Supplementary Tables S1 and S2. One hundred and eighteen genes were more highly expressed in the asymptomatic inflorescence tissue, and 150 genes were more highly expressed in the choke stroma tissue. Numerous as yet uncharacterized genes were among the differentially expressed genes, 45 more highly expressed in the choke stroma tissue and 32 more highly expressed in the inflorescence tissue. There were also numerous differentially expressed genes that could be identified by BlastX matches to a type of enzyme or protein, but the specific substrate or function is not known.

### 3.3. Differential Expression of E. festucae Genes Possibly Involved in Stroma Development

A five-gene cluster was proposed to function in the production of an as yet unidentified secondary metabolite that may regulate stroma development [50,51]. In the *E. typhina*/*D. glomerata* and *E. elymi*/*Elymus* sp. symbioses, the five genes of the cluster were up-regulated in stroma tissue relative to asymptomatic inflorescence tissue [50]. In the *E. festucae*/strong creeping red fescue interaction analyzed here, two of the five genes in the cluster, *fxbA* (EfM3.019620) and *mfsB* (EfM3.019610), were up-regulated in the choke stroma tissue relative to the asymptomatic inflorescence tissue (Table 5). This expression pattern is consistent with the model presented by Berry [50], in which the activity of IrlA on an as yet unidentified compound results in a non-stroma-producing product, but the competing activity of FxbA on the precursor compound leads to a stroma-producing compound that is transported out of the cell by MfsB. Over expression of *fxbA* in the choke stroma tissue relative to the asymptomatic inflorescence tissue could increase the rate of conversion of the putative precursor to the stroma-producing compound relative to the non-stroma-producing compound.

Candidate host specialization genes have been identified and hypothesized to be involved in choke stroma formation [52]. Host specialization among *Epichloë* spp. is considered to involve secreted proteins [35,52]. Comparative genome scans of *E. typhina* subsp. *typhina* and *E. typhina* subsp. *clarkii* identified five candidate host specialization-secreted proteins based on high dN/dS ratios, indicative of positive selection [52]. Three of these candidates encode enzymes that could directly interact with the host grass—a pectin methylesterase (EfM3.008730), a peroxidase (EfM3.007770), and an endo-1-4-beta-xylanase (EfM3.040190). One of the host specificity candidates is a small-secreted protein (EfM3.008740) also identified as a candidate effector protein [35]. One is an unannotated gene that has a cyanovirin-N domain. Schirrmann et al. hypothesized that the three enzymes may be involved in facilitating stroma formation [52]. In the transcriptome comparison presented here, the pectinesterase and endo-1-4-beta-xylanase were differentially expressed, but were more highly expressed in asymptomatic inflorescence tissue (Supplementary Table S3) than choke stroma tissue. The other host specialization candidates were not significantly differentially expressed.

### 3.4. Differential Expression of E. festucae Genes Possibly Involved in Plant–Fungal Interactions

The interaction of fungal plant pathogens and symbionts with their hosts involves effector proteins, characterized as small-secreted proteins that can be important for colonization or for evasion of host defenses [53,54]. *E. festucae* expresses numerous small-secreted proteins that may function as effectors in its interaction with the host grass [31]. However, none of these small-secreted proteins have been functionally confirmed as effectors. Hassing et al. analyzed the *E. festucae* genome sequence for potential effectors and identified 141 candidate genes [35]. Some of these candidate effector genes were differentially expressed between the choke stroma tissue and the asymptomatic inflorescence tissue. Six were more highly expressed in the choke stroma tissue and 19 were more highly expressed in the asymptomatic inflorescence tissue (Supplementary Tables S1 and S2). The change in expression between the asymptomatic inflorescence tissue and the choke stroma tissue of these candidate effectors suggests that they may affect a change in interaction with the host that likely occurs in the switch from asymptomatic to macroscopic growth of the fungus.

Another gene that was overexpressed in the choke stroma tissue is EfM3.028290, which encodes a small-secreted protein similar to cerato-platanins. Cerato-platanin genes are found in many fungal genomes and appear to be unique to fungi [55]. The name originates from the first described member of this family, which was isolated from the fungal pathogen *Ceratocystis fimbriata* f. sp. *platani* and was found to be phytotoxic [56]. Similar proteins have since been characterized from other fungi and have been reported to have numerous effects such as phytotoxicity, elicitation of plant defense responses, and virulence [56,57]. Cerato-platanin gene expression from *C. platani* was higher during rapid hyphal growth and spore formation [58]. Based on the similarity in protein structure of cerato-platinin and plant expansins, Baccelli proposed that the cerato-platanins may function in loosening of fungal and plant cell walls, facilitating hyphal elongation during growth, as well as aiding in release of nutrients from plant cells [55]. The *E. festucae* cerato-platanin-like protein has not been functionally characterized, but the reported features of similar proteins may be relevant to what is seen in choke disease, where the fungal growth rate is increased and the encased plant tissue is expressing more defensive proteins (discussed more below).

### 3.5. Differential Expression of E. festucae CAZymes, Transport, and Lipid Metabolism Genes

Genes encoding carbohydrate-active enzymes (CAZymes) were among the differentially expressed genes. CAZymes play a major role in breaking down, modifying, or synthesizing polysaccharides. Fungi often produce CAZymes to degrade plant cell walls, and plant pathogenic fungi generally possess more CAZyme genes than saprophytic or symbiotic fungi [59]. Twenty-three genes in the auxiliary activities (AA), carbohydrate esterases (CE), glycoside hydrolases (GH), glycosyltransferases (GT), and carbohydrate-binding modules (CBM) classes were differentially expressed (Table 6).

Differential expression of some of the CAZymes is likely relevant to the difference in fungal growth between the two tissues. Genes for two secreted proteins, beta-fructofuranosidase and alpha-l-arabinofuranosidase, that are likely involved in accessing host-produced carbohydrates were more highly expressed in the choke stroma tissue. Expression of beta-fructofuranosidase (EfM3.074710), a secreted invertase, was significantly higher in the choke tissue. Secreted invertase hydrolyzes apoplastic sucrose, which would likely be abundant in the inflorescence tissue [60]. This result is consistent with previous research that detected high invertase activity in choke stroma tissue of Chewings fescue and strong creeping red fescue [17]. The invertase activity produced by *E. festucae* grown in culture was shown to be sucrose-inducible rather than glucose-repressible [17]. The expression of a cytoplasmic invertase (EfM3.072380) in this study did not differ between the two tissues. 

Alpha-l-arabinofuranosidase (EfM3.015180) catalyzes the hydrolysis of terminal alpha-l-arabinofuranosidic bonds in hemicelluloses, such as arabinoxylan and l-arabinan, which are present in host cell walls, releasing l-arabinose [61,62]. In culture, *E. festucae* isolates grew as well on arabinose as on sucrose [63]. The up-regulation of both beta-fructofuranosidase and alpha-l-arabinofuranosidase in the choke stroma tissue suggests an increase in sucrose and arabinose utilization, which agrees with the hypothesis that the ability to utilize sugars and grow rapidly is critical for stroma formation [16,63].

In contrast to the up-regulation of alpha-l-arabinofuranosidase, genes for two other plant cell wall degrading enzymes were down-regulated in the stroma tissue—a pectin methylesterase gene (EfM3.008730) and an endo-1,4-beta-xylanase (EfM3.040190). Both of these genes are candidate host specialization genes [52], discussed above. The interactions of the fungal endophyte with the host plant cell wall are apparently different in the asymptomatic inflorescence tissue and the choke stroma tissue.

Expression of two genes involved in the synthesis of chitin and glucan, important fungal cell wall components, were up-regulated in choke stroma tissue. A chitin synthase (EfM3.049120) and a beta-1,3-glucan synthase (EfM3.026320) were up-regulated in the choke stroma tissue relative to the asymptomatic inflorescence tissue. Increased glucan and chitin synthesis would be expected during the proliferative growth of *E. festucae* stroma tissue, which envelops the developing inflorescence.

Other CAZymes were down-regulated in the choke stroma tissue, being more highly expressed in the asymptomatic inflorescence tissue. A chitinase (EfM3.024310) [38] and a beta-1,6-glucanase (EfM3.013890) [40], both hydrolytic enzymes that act on fungal cell walls, were down-regulated in the choke stroma tissue. The down-regulation of these fungal cell wall hydrolytic enzymes, combined with the up-regulation in the fungal cell wall synthesis enzymes, is possibly a factor in the increased growth and biomass accumulation of fungal tissue in the choke stroma tissue. However, a glycosylphosphatidylinositol (GPI)-anchored beta-1,3-endoglucanase (EfM3.044280) was more highly expressed in the choke stroma tissue. A potential role of this protein is to degrade the plant cell wall [64], but it may also function to modify its own wall, as was reported for a similar enzyme in *Aspergillus nidulans* [65]. 

Another CAZyme protein that may be important in the interaction of *E. festucae* with the host grass was a secreted protein with two LysM domains (EfM3.029340) that was more highly expressed in the asymptomatic inflorescence tissue than in the choke stroma tissue. Transcripts for this LysM domain-containing protein were also reported to be abundant in *E. festucae*-infected leaf sheath tissue [31]. In the asymptomatic association of *E. festucae* with the host grass, the chitin in intercellular hyphae does not stain with wheat germ agglutinin, indicating that it is somehow masked, presumably as a defense mechanism to avoid being degraded by plant chitinase [66]. *E. festucae* can penetrate the leaf cuticle and emerge from the interior of the plant, forming an epiphytic hyphal network [67]. The epiphytic hyphae do stain, indicating the chitin masking agent is no longer associated with the hyphae. Here, transcripts for the LysM domain-containing protein were down-regulated in choke stroma tissue, an external tissue like the epiphytic leaf surface hyphae. In other fungal species, secreted LysM domain effector proteins have been shown to bind chitin and thereby suppress the chitin-triggered plant defense against fungal pathogens [68,69,70]. This suggests a potential role of this protein in maintaining the symbiotic interaction in internal asymptomatic tissue by preventing a chitin-triggered plant defense response.

In addition to the highly expressed aquaglyceroporin (EfM3.025350) discussed above, there were several genes involved in transport that were differentially expressed between the two tissue types (Supplementary Table S4). Genes involved in sugar, amino acid, and oligopeptide transport were among those more highly expressed in the choke stroma tissue, suggesting a higher demand for nutrient uptake in the choke stroma tissue. Major facilitator superfamily (MFS) transporters and ATP-binding cassette (ABC) transporters were also among the differentially expressed transport-related genes, but the compounds transported for many of these genes are not yet known. Some of these transporter genes were up-regulated in the choke stroma tissue and some were down-regulated. The differential expression of transport-related genes between the choke stroma tissue and the asymptomatic inflorescence tissue indicates differences in how the two tissue types interact with their environment. 

There were several lipid metabolism genes that were up-regulated in the choke stroma tissue, suggesting that there may be a change in lipid composition in stroma tissue relative to the intercellular fungal tissue. The biosynthetic pathway for glucosylceramide, a sphingolipid, is known, and all the genes have been functionally characterized in other fungal species. Four of the eight genes for the biosynthesis of glycosylceramide were significantly more highly expressed in the choke stroma tissue (Table 7). Glucosylceramide is a component of the plasma membranes, and is also found in exosomes [71]. Glucosylceramide can alter the properties of the plasma membrane, favoring hyphal growth [72], which is clearly enhanced in the choke stroma tissue.

Other lipid metabolism genes overexpressed in the choke stroma tissue are phosphatidic acid phosphatase (EfM3.009180), fatty acyltransferase (EfM3.017140), long-chain fatty acid CoA ligase (EfM3.034500), phospholipid-translocating P-type ATPase (EfM3.040210), phosphatidylserine decarboxylase (EfM3.054610), linoleate (8R)-dioxygenase (EfM3.057040), aminophospholipid translocase (EfM3.073880), and hydroxyacyl-CoA dehydrogenase-like (EfM3.074020).

### 3.6. Differential Expression of E. festucae Secondary Metabolite Biosynthetic Genes

Genes encoding alkaloid biosynthetic enzymes were down-regulated in the choke stroma tissue. The fungal production of antiherbivore alkaloids is a common feature of many of the *Epichloë*/host grass interactions. Because of the commercial importance of the alkaloids to forage grasses, their structures, biosynthesis, and genes have been topics of intensive investigation [78]. There are four main categories of alkaloids produced by *Epichloë* endophytes: The indole-diterpenes, lolines, ergot alkaloids, and peramine. There is considerable variation among *Epichloë* spp. and isolates within a species in the biosynthesis of particular alkaloids, generally due to variation in the presence or absence of particular genes among isolates of a species [78]. Stroma-forming endophyte species generally produce lower levels of alkaloids compared to seed-transmitted (asexual) species [79,80]. Alkaloid gene expression can also vary depending on the plant tissue. Alkaloid genes of *E. coenophila*, a non-stroma-forming endophyte of tall fescue, were significantly down-regulated in the ovaries compared with pseudostems [33]. 

In the *E. festucae*/strong creeping red fescue interaction studied here, there was a change in the fungal metabolism between the asymptomatic growth within the inflorescence tissue to the choke stroma tissue regarding expression of alkaloid biosynthetic genes. Transcripts for the gene for peramine biosynthesis and for six out of the 11 genes involved in ergot alkaloid biosynthesis were significantly down-regulated in the choke stroma tissue as compared to the asymptomatic inflorescence tissue, although for some, the RPKM (reads per kilobase of exon model per million mapped reads) values were low (Table 8). Some of these genes were not included in the differential expression table (Supplementary Table S1) because their expression did not meet the filter threshold of 10 RPKM. Sequences for the genes involved in indole-diterpene and loline biosynthesis were not detected in either the asymptomatic inflorescence or the stroma tissue, raising the possibility that these genes are not present in this isolate of *E. festucae*. Variation in alkaloid synthesis capability among *E. festucae* isolates has previously been reported [81].

Peramine biosynthesis is carried out by a multifunctional non-ribosomal peptide synthetase, encoded by the *perA*-1 gene. A partially deleted *perA* gene, designated *perA*-2, is present in many *Epichloë* spp. isolates, including some *E. festucae* isolates infecting strong creeping red fescue [78,81]. Peramine is not detected in grasses infected with an *Epichloë* isolate containing the *perA*-2 allele, although gene expression may be detected, leading to the speculation that perhaps a different secondary compound is produced from this mutated allele [81]. Berry et al. reported that the *perA*-2 allele encodes a functional diketopiperazine synthetase [82]. Here, *perA* transcripts were detected, but a 17 bp insert sequence common to the *perA*-2 allele [83] was present, indicating that this *E. festucae* isolate carries the variant allele. The same mutation generating the *perA*-2 allele is found in many *Epichloë* isolates, suggesting it had a single origin [83]. Peramine biosynthesis, both in culture and in planta, could be recovered in an *Epichloë* Lp-TG3 isolate harboring a *perA*-2 gene through *Agrobacterium*-mediated transformation with a *perA*-1 gene [84].

In an *E. festucae*/meadow fescue (*Lolium pratense*) interaction, no lolines were detected in choke stroma tissue but were detected in asymptomatic inflorescences; RNA-Seq analysis indicated that the loline biosynthetic genes were down-regulated in the stroma tissue [85]. The authors considered that down-regulation of loline levels in choke stroma tissue was important in avoiding damaging the *Botanophila* flies that are required for completion of the sexual cycle of *E. festucae*. Stroma-bearing tillers were reported to experience greater herbivory damage from arthropods than non-stroma-bearing tillers [80]. Apparently, the shift in the fungal growth habit from the asymptomatic growth within the plant tissue to the visible stroma results in down-regulation of alkaloid genes in general, as seen here in this *E. festucae*/strong creeping red fescue interaction.

Other secondary metabolite biosynthetic genes were also down-regulated in the choke stroma tissue. In addition to the *perA* alleles, there are other non-ribosomal peptide synthetase genes in the *Epichloë* spp. genomes. Non-ribosomal peptide synthetases are large multifunctional proteins involved in synthesis of a diverse range of bioactive compounds [86]. Johnson et al. examined 20 *Epichloë* spp. and identified 12 NRPS genes, although not all were present in all isolates [87]. Here, one of the previously identified fungal non-ribosomal peptide synthetases, NRPS7 (EfM3.081210), was overexpressed in the asymptomatic inflorescence tissue (Table 9). The *E. festucae* 2368 genome sequence (http://csbio-l.csr.uky.edu/ef2011/) was also searched for genes annotated as non-ribosomal peptide synthases, and two additional genes were identified that were overexpressed in the asymptomatic inflorescence tissue. One non-ribosomal peptide synthetase appears to be from the two annotated genes EfM3.059650 and EfM3.059660. These two annotated genes are likely actually a single gene, since they are adjacent to each other and both are most similar to different regions of the same *Lecanicillium* sp. (Cordycipitaceae) gene encoding the synthesis of verlamelin, an antifungal compound [88]. 

In addition to the synthesis of secondary metabolites by non-ribosomal peptide synthetases, many bioactive compounds are produced by polyketide synthases [89]. The *E. festucae* 2368 genome sequence (http://csbio-l.csr.uky.edu/ef2011/) contains 17 genes that are annotated as polyketide synthases, the functions of which are not yet known. Most were not differentially expressed, but two were more highly expressed in the asymptomatic inflorescence tissue than in the choke stroma tissue (Table 9). One of the polyketide synthases (EfM3.081190) is part of a gene cluster with two of the non-ribosomal peptide synthetases (EfM3.081210 and EfM3.081180) that were also more highly expressed in the asymptomatic inflorescence tissue. None of the differentially expressed *E. festucae* non-ribosomal peptide synthetases or polyketide synthases have yet been characterized as to the compounds they produce.

Coordinately regulated gene clusters are often involved in biosynthesis of secondary compounds. In addition to differential expression of two genes from the five-gene cluster proposed by Berry [50] to be involved in stroma formation (discussed above), genes from other gene clusters were also differentially expressed between the two tissue types. Transcripts from a cluster of four genes, EfM3.029270 to EfM3.029300, were more highly expressed in the choke stroma tissue than the asymptomatic inflorescence tissue, with two of them, EfM3.029280 and EfM3.029300, being among the 25 most highly expressed fungal genes in the choke stroma tissue. EfM3.029270 encodes a subtilisin-like protease designated *Efe*-PrtL [90]. The other three genes in the cluster encode as yet uncharacterized proteins. This gene cluster appears to be restricted to *E. festucae*, (accessions ADFL02000205.1, NRIB01000040.1, AFRX02000601.1, NDBD01002901.1), *E. mollis* (accession JFGW01000214.1), and some isolates of *E. bromicola* (accessions NRIC01000304.1, JFHA01000306.1), since it is not found in other *Epichloë* spp. for which whole genome sequences are available. Although in this study some of the genes of the cluster are highly and differentially expressed in stroma tissue, it is questionable whether they are critical to stromata production, since this gene cluster is not found in some *Epichloë* spp. that produce stromata, such as *E. typhina* subsp. *typhina* infecting *D. glomerata* (accession SRX2830678) and *E. typhina* subsp. *clarkii* infecting *Holcus lanatus* (accession SRX2830679).

Two genes from another gene cluster were more highly expressed in the choke stroma tissue relative to the asymptomatic inflorescence tissue. EfM3.014890 (dipeptidase) and EfM3.014910 (MFS transporter) are members of a gene cluster similar to the epipolythiodiketopiperazine gene cluster in *Claviceps purpurea* [91]. The cluster consists of 11 genes (EfM3.014870 to EfM3.014970) and all were expressed in both tissue types, with only the dipeptidase and the MFS transporter being differentially expressed. In the *C. purpurea* strain analyzed, the cytochrome P450 enzyme of the cluster was dysfunctional and no epipolythiodiketopiperazine was produced, but new, previously unknown metabolites were produced [91]. Such compounds have not been reported in *Epichloë* spp., but the expression of genes from this cluster suggests they may be present. 

All genes of an apparent five-gene cluster (EfM3. 057200 to EfM3.057240) were down-regulated in the choke stroma tissue relative to the asymptomatic inflorescence tissue. However, the specific functions of the encoded proteins are not known. This gene cluster is another cluster with limited presence among the sequenced *Epichloë* genomes, being present only in *E. festucae* and *E. typhina* subsp. *poae*.

### 3.7. Expression of Previously Identified E. festucae Symbiotic-Related Genes

Several *E. festucae* genes have been reported that are critical to the maintenance of a stable symbiosis in the asymptomatic tissues of the host plant. For many of these genes, a knock-out resulted in unregulated fungal growth, leading to increased fungal biomass and stunting of the host (Supplementary Table S5). These are features similar to what happens in choke disease, although none of the symbiosis-related gene knock-outs resulted in the fungal mycelium becoming macroscopic. However, none of these symbiosis-related genes were significantly differentially expressed between the choke stroma tissue and the asymptomatic endophyte-infected inflorescence tissue. The lack of differential expression of the symbiosis-related genes between the two tissue types was unexpected, since many of these genes are involved in regulating the restricted growth of the fungus in the asymptomatic tissues [92].

Eaton et al. reported a transcriptome comparison of *E. festucae*/*Lolium perenne* associations of wild-type and knock-out lines of three of the symbiosis-related genes: A component of the NADPH oxidase complex *noxA*, a stress-activated mitogen-activated protein kinase *sakA*, and a transcription factor *proA* [93]. They identified a core set of 182 genes that were differentially expressed in all three knock-out lines that were considered to be contributing to the antagonistic nature of the mutant lines. Although development of a choke stroma could also be considered as an antagonistic interaction, only 22 of the 182 core genes were differentially expressed between the choke stroma tissue and the asymptomatic inflorescence tissue. Of the differentially expressed genes, only 13 of the 22 were in the same direction as reported for the three knock-out lines (Supplementary Table S6). Despite sharing some features in common, the gene expression profiles of the antagonistic mutants and the choke stroma tissue are substantially different.

### 3.8. Plant Genes Differentially Expressed Between the Asymptomatic Inflorescence and the Choke Stroma Tissues

There were dramatic changes in plant gene expression between the asymptomatic inflorescence and choke stroma tissues. There were a total of 7826 differentially expressed plant genes meeting the criteria of a log_2_ fold change >2 (adjusted *p* < 0.01), representing over 10% of the total assembled plant transcriptome contigs. There were 2964 genes more highly expressed in the choke stroma tissue and 4862 genes were more highly expressed in the asymptomatic inflorescence tissue. A full list of differentially expressed plant genes is presented in Supplementary Table S7. Several over-represented biological process categories were identified among differentially expressed genes in both tissue types by Blast2GO analysis. The over-represented GO terms of the differentially expressed plant transcripts are summarized in Table 10, only showing the most specific GO terms. Genes related to carbohydrate metabolic process and transport, which includes small molecule transport between and within cells, were over-represented in the choke stroma tissues, indicating basic metabolic differences between the tissues. Genes related to biotic and abiotic stresses were also up-regulated in the choke stroma tissue (discussed more below), whereas genes related to reproductive development, including the GO categories of cell cycle, flower development, and cell differentiation, were down-regulated. The down-regulation of genes in these processes suggested the normal reproductive process was interrupted in the choke stroma tissue, which would suppress seed development. Choke disease has been reported to significantly reduce seed yields [10,14,17].

Of particular interest regarding the endophyte—plant interaction is the over-representation of genes in the GO categories of response to biotic stimulus (46 genes) and response to water deprivation (6 genes) in the choke stroma tissue (Table 11). This suggests that the host strong creeping red fescue plant responded to *E. festucae* during choke stroma development as if the endophyte was a pathogen. Apparently, the plant was sensing the presence of the fungal endophyte as being antagonistic and responded by expressing defensive genes. As discussed above, an endophyte gene encoding a cerato-platanin-like protein (EfM3.028290), that in other species was found to induce plant defense responses, was over-expressed in the choke stroma tissue.

Among the plant proteins overexpressed in the choke stroma tissue in the GO category response to biotic stimulus were several pathogenesis-related (PR) proteins—a class 1 chitinase (PR-3), PR-4, thaumatin-like proteins (PR-5), peroxidases (PR-9), PR-10, and lipid transfer proteins (PR-14). These proteins have all been associated with antifungal activity [94,95,96,97]. PR-10 was previously reported in *E. festucae*-infected ryegrass vegetative tissues [44]. 

Transcripts of 12 non-specific lipid-transfer proteins were among the genes in the GO category of response to biotic stimulus that were overexpressed in the choke stroma tissue. Non-specific lipid-transfer proteins are a family of small cysteine-rich proteins involved in various biological functions, including resistance to biotic and abiotic stresses [98,99,100]. Five of the 12 over-expressed lipid transfer proteins were similar to the DIR1 (defective in induced resistance)-type lipid-transfer proteins. The *A. thaliana* lipid-transfer protein DIR1 has been shown to be critical for the development of systemic acquired resistance [101]. Based on its structure, DIR1 was considered to be a specific category of lipid-transfer protein [102]. We detected eight putative DIR1-like lipid-transfer protein sequences in the strong creeping red fescue transcriptome, five of which were up-regulated in the choke stroma tissue. 

Other proteins in this GO category over-expressed in the choke stroma tissue were calcium-dependent protein kinases, lectin-domain containing receptor kinases, probable leucine-rich repeat (LRR) receptor-like serine/threonine-protein kinases, triacylglycerol lipases, 1-aminocyclopropane-1-carboxylate oxidase, and an ethylene-responsive transcription factor. The over-represented GO response to water deprivation suggests that the plant was also under abiotic stress during development of the choke stroma. Six up-regulated genes in the response to water deprivation GO category supported the findings of White et al. that transpiration is enhanced in stromal leaves [49]. Those six genes encode a late embryogenesis abundant protein, a putative ornithine aminotransferase, a dehydrin, a heat shock protein, an aquaporin, and a histidine kinase. Moisture from the plant transpiration stream likely plays an important role in the development of the stroma [49]; they found that the epidermal cells of the stromal leaves were damaged, leading to increased evaporation from the surface. Transpirational water loss through the damaged stromal leaf may result in water deprivation in the plant tissue within the stroma.

## 4. Conclusions

Here, we compared gene expression of both the fungal endophyte, *E. festucae*, and the host plant, strong creeping red fescue, between asymptomatic inflorescence tissue and choke stroma tissue. Hundreds of fungal genes were differentially expressed between the two tissue types, supporting the expectation of a change in fungal metabolism occurring in the change from macroscopically asymptomatic to symptomatic growth.

Fungal genes overexpressed in the choke stroma tissue indicate a change in fungal carbohydrate and lipid metabolism relative to the asymptomatic state. Also, over-expressed in the choke stroma tissue were genes encoding enzymes of fungal cell wall synthesis, which would support the generation of the enhanced mycelial mass of the stroma. Several candidate effector genes [35] were differentially expressed between the two tissue types. Since such genes may be involved in the interaction of *E. festucae* with its host, the identified differentially expressed candidate effector genes are good targets for future functional characterization, including the use of gene knock-outs. Most of the alkaloid biosynthetic genes and genes encoding some previously identified secreted hydrolytic enzymes were down-regulated in the choke stroma tissue. Somewhat surprisingly, none of the numerous genes previously identified as critical to the asymptomatic symbiosis of the endophyte and host were differentially expressed between the two tissues. In future studies, it would be interesting to microscopically examine apparently asymptomatic inflorescence tissue to determine if there were any microscopic signs of enhanced fungal growth that did not result in formation of a macroscopic stroma. 

Over 7000 plant genes were differentially expressed between the choke stroma tissue and the asymptomatic inflorescence tissue. In contrast to the numerous differentially expressed plant genes reported here, in transcriptome comparisons of endophyte infected with endophyte-free tall fescue inflorescence tissues, Nagabhyru et al. reported almost no significant difference in plant gene expression profiles [33]. Similarly, in comparisons of endophyte-infected and endophyte-free vegetative tissues of strong creeping red fescue, perennial ryegrass, and tall fescue, only modest changes in plant gene expression have been reported [31,32,34]. The criterion we used for differential expression, a fold change greater than 4, is more stringent than the previous studies which used a fold change greater than 2. That there were so many differentially expressed plant genes is an indication of a large difference in plant metabolism in the choke stroma relative to the asymptomatic tissues. The plant tissue within the stroma is a developmentally arrested inflorescence, and so gene expression would be expected to differ from that of a normally developed inflorescence. Also, the higher expression of the cerato-platanin-like gene and numerous plant pathogenesis-related defense genes suggests that plant cells may be dying during stroma formation, which would also contribute to differences in the plant transcriptome relative to the asymptomatic inflorescence tissue.

Plant genes over-expressed in the choke stroma tissue included genes related to water and biotic stress. The over-expression of biotic stress genes indicates the plant was responding to the fungal endophyte as if it was a pathogen by inducing expression of defense-related genes. The plant tissue was also apparently experiencing water stress, which may be attributable to an increased diversion of available water to the developing stroma tissue. Plant genes related to reproductive processes were down-regulated in the stroma tissue, likely due to the interference with seed development by the physically restrictive growth of the stroma. Future studies could include transcriptome analyses of a developmental time series of both macroscopically asymptomatic inflorescence tissues, as well as stroma tissues. It would be interesting to determine if the lack of stroma development in the asymptomatic inflorescence tissues is due to increased expression of plant defense genes at an early developmental stage.

This study provides a step towards ultimately understanding what triggers the initiation of stroma development in some *Epichloë* spp. What triggers the initial shift in fungal growth habit in choke stroma development is not yet known, but presumably is related to a host metabolic change that occurs in the transition from vegetative tissue to inflorescence development. Another complementary question for future research is why in Type 2 interactions [13] on the same plant experiencing the same environmental conditions do some inflorescences develop stromata and some do not. Also, it would be interesting to examine the evolution of non-stroma-forming *Epichloë* isolates from stroma-forming *Epichloë* isolates. *E. festucae* isolates infecting *F. rubra* often cause choke disease, whereas *E. festucae* isolates infecting *F. trachyphylla* (hard fescue) and *Lolium perenne* (perennial ryegrass) do not. *E. festucae* isolates from *Festuca* spp. are closely related to the *E. festucae* var *lolii* isolates from perennial ryegrass, but are even more closely related to the proposed new taxon, *Lp*TG-3, also from *L. perenne* [103]. Knowledge of these phylogenetic relationships will be important in guiding future research on development of choke disease.

## Figures and Tables

**Table 1:** Strong creeping red fescue transcriptome assembly.

No. of Contigs	75,264
Average length (bp)	516
Min/Max length (bp)	200/8421
N50 (bp)	322
Total assembly length (bp)	38,860,733

**Table 2:** RNA-seq mapping data.

Tissue Type		Total Reads	Fungal Mapped Reads, %	Plant Mapped Reads, %
Choke stroma	Replicate 1	124,013,190	82.93	9.09
	Replicate 2	131,631,954	76.30	12.00
	Replicate 3	188,024,471	76.04	14.23
Asymptomatic inflorescence	Replicate 1	95,854,782	1.19	68.20
	Replicate 2	102,612,960	1.04	68.08
	Replicate 3	149,249,658	1.55	69.88

**Table 3:** The 25 most abundant *Epichloë festucae* transcripts in asymptomatic strong creeping red fescue inflorescence tissue.

Gene Model and Protein Annotation		RPKM ^a^	Log_2_ FC ^b^	FDR *p*
1	EfM3.056300 Ubiquitin―40S ribosomal protein S27a fusion	68,394	−8.2	0
2	EfM3.043630 Polyubiquitin	16,619	−5.6	8.1 × 10^−13^
3	EfM3.021690 Translation elongation factor EF-1 alpha	12,254	−4.4	4.4 × 10^−8^
4	EfM3.005710 Ubiquitin―60S ribosomal protein L40 fusion	11,137	−5.7	1.0 × 10^−11^
5	EfM3.046430 Candidate effector protein [31,35]	10,899	−6.2	0
6	EfM3.020500 Candidate effector protein [31,35]	4532	−12.0	0
7	EfM3.018170 Uncharacterized; NC12 [36]	3799	−8.0	0
8	EfM3.028480 GigA/NC25 [37]; cyclic peptide precursor	3421	−6.6	0
9	EfM3.025530 Glucose repressible protein, Grg1 [31]	2549	NS	NS
10	EfM3.029340 LysM domain containing protein [31]	2380	−5.1	4.3 × 10^−10^
11	EfM3.072910 Uncharacterized small protein	1672	NS	NS
12	EfM3.057230 Candidate effector protein [35]	1398	−6.7	0
13	EfM3.067730 Candidate effector protein [31,35]	1360	−3.6	4.8 × 10^−8^
14	EfM3.024310 Chitinase [38]; glycosyl hydrolase family 18	1106	−3.9	5.1 × 10^−7^
15	EfM3.046900 Glyceraldehyde 3-phosphate dehydrogenase	1098	−2.9	3.7 × 10^−7^
16	EfM3.006460 Candidate effector protein [35]	1004	−17.1	0
17	EfM3.026500 40S ribosomal protein S17	948	−3.0	3.3 × 10^−4^
18	EfM3.028690 Secreted serine/threonine-rich protein	931	NS	NS
19	EfM3.050840 Candidate effector protein [35]	828	−8.4	8.6 × 10^−10^
20	EfM3.063660 Antifungal protein, candidate effector protein [31,35,39]	815	−3.7	5.8 × 10^−10^
21	EfM3.013890 Beta-1,6-glucanase [40,41]	721	−5.4	5.5 × 10^−12^
22	EfM3.030230 Candidate effector protein [35]	641	−14.9	0
23	EfM3.031590 60S ribosomal protein L27a	615	NS	NS
24	EfM3.073190 ADP-ribosylation factor	603	−2.2	0.007
25	EfM3.019720 Clock-controlled protein-6; Mmc	598	NS	NS

^a^ RPKM value (reads per kilobase of exon model per million mapped reads) is the mean of the three asymptomatic inflorescence sequence replicates. ^b^ Fold change (FC) is the ratio of the largest mean RPKM values to the lowest mean RPKM values in the comparison of choke stroma tissues with asymptomatic inflorescence tissues. A negative fold change value indicates the gene was more highly expressed in the asymptomatic inflorescence tissue. NS indicates that there was no statistical difference in gene expression between the asymptomatic inflorescence and stroma tissues at the false discovery rate (FDR)-adjusted *p*-value < 0.01. The statistical model used by the analysis program corrects for differences in library size, so the fold changes cannot be determined by simple algebraic calculations of the mean RPKM values of the two tissue types.

**Table 4:** The 25 most abundant *Epichloë festucae* transcripts in strong creeping red fescue choke stroma tissue.

Gene Model and Protein Annotation		RPKM ^a^	Log_2_ FC ^b^	FDR *p*
1	EfM3.025530 Glucose repressible, Grg1	34,478	NS	NS
2	EfM3.072910 Uncharacterized small protein	7962	NS	NS
3	EfM3.019720 Clock-controlled protein-6; Mmc	6484	NS	NS
4	EfM3.029300 Uncharacterized protein	5815	3.8	7.0 × 10^−5^
5	EfM3.007740 Candidate effector protein [35]	3865	5.0	1.9 × 10^−4^
6	EfM3.075190 Candidate effector protein [35]	2779	4.9	4.5 × 10^−5^
7	EfM3.059820 Ferritin/ribonucleotide reductase-like	2428	3.2	0.002
8	EfM3.021690 Translation elongation factor EF-1 alpha	2340	−4.4	4.4 × 10^−8^
9	EfM3.025350 Aquaglyceroporin	2241	3.7	5.2 × 10^−4^
10	EfM3.059410 Pyruvate/phosphoenolpyruvate kinase	2223	4.4	2.9 × 10^−4^
11	EfM3.022140 Histone H3	2155	NS	NS
12	EfM3.003250 Uncharacterized; coiled-coil domain	2075	NS	NS
13	EfM3.043090 Hydrophobic surface binding protein	1760	NS	NS
14	EfM3.031680 Candidate effector protein [35]	1720	5.4	1.9 × 10^−5^
15	EfM3.079420 Candidate effector protein [35]; Hydrophobin	1539	6.4	1.9 × 10^−4^
16	EfM3.030930 RhgA [42], secreted	1520	NS	NS
17	EfM3.019650 EsdC [43], glycogen-binding domain	1480	NS	NS
18	EfM3.064250 Uncharacterized	1458	4.5	1.3 × 10^−5^
19	EfM3.029280 Vacuolating cytotoxin domain protein	1433	5.1	1.2 × 10^−4^
20	EfM3.064240 Uncharacterized	1399	3.3	1.3 × 10^−5^
21	EfM3.043630 Polyubiquitin	1357	−5.6	8.1 × 10^−13^
22	EfM3.045575 Autophagy protein Apg5	1252	NS	NS
23	EfM3.075710 Cu/Zn-superoxide dismutase [44]	1249	NS	NS
24	EfM3.028690 Secreted serine/threonine-rich protein	1160	NS	NS
25	EfM3.017130 Candidate effector protein [35], phospholipase	1156	3.9	1.7 × 10^−4^

^a^ RPKM value (reads per kilobase of exon model per million mapped reads) is the mean of the three choke stroma tissue sequence replicates. ^b^ Fold change (FC) is the ratio of the largest mean RPKM values to the lowest mean RPKM values in the comparison of choke stroma tissues with asymptomatic inflorescence tissues. A positive fold change value indicates that the gene was more highly expressed in the choke stroma tissues, and a negative fold change value indicates the gene was more highly expressed in the asymptomatic inflorescence tissue. NS indicates that there was no statistical difference in gene expression between the asymptomatic inflorescence and stroma tissues at the false discovery rate (FDR)-adjusted *p*-value < 0.01. The statistical model used by the analysis program corrects for differences in library size, so the fold changes cannot be determined by simple algebraic calculations of the mean RPKM values of the two tissue types.

**Table 5:** Expression of genes of the five-gene cluster proposed by Berry [50] to be involved in regulation of stroma development.

Gene (Gene Model)	RPKM ^a^	Log_2_ FC ^b^	FDR *p*-Value
*pdtA* (EfM3.019580)	45	NS	NS
*afrA* (EfM3.019590)	33	NS	NS
*irlA* (EfM3.019600)	630	NS	NS
*mfsB* (EfM3.019610)	226	4.5	0.0003
*fxbA* (EfM3.019620)	147	5.0	1.1 × 10^−5^

^a^ RPKM value (reads per kilobase of exon model per million mapped reads) is the mean of the three choke stroma tissue sequence replicates. ^b^ Fold change (FC) is the ratio of the largest mean RPKM values to the lowest mean RPKM values in the comparison of choke stroma tissues with asymptomatic inflorescence tissues. A positive fold change value indicates that the gene was more highly expressed in the choke stroma tissues. NS indicates that there was no statistical difference in gene expression between the asymptomatic inflorescence and stroma tissues at the false discovery rate (FDR)-adjusted *p*-value < 0.01. The statistical model used by the analysis program corrects for differences in library size, so the fold changes cannot be determined by simple algebraic calculations of the mean RPKM values of the two tissue types.

**Table 6:** Differential gene expression in CAZyme genes at false discovery rate-adjusted *p* < 0.01.

CAZymes	Gene Model	Predicted Function	Log_2_ FC ^a^
AA11	EfM3.037810_1	Glycoside hydrolase	3.6
AA5	EfM3.061080_1	WSC domain	13.2
AA7	EfM3.019620_1	*Efe*-FxbA; FAD-binding oxidoreductase	5.0
CE16	EfM3.017140_1	Fatty acyltransferase	2.9
CE3	EfM3.073810_1	Carbohydrate esterase family 3, secreted	−4.2
CE8	EfM3.008730_1	Pectinesterase, secreted	−3.1
GH10	EfM3.040190_1	Endo-1,4-beta-xylanase, secreted	−3.5
GH125	EfM3.071270_1	DUF1237 family protein, secreted	−2.4
GH16	EfM3.039700_1	Fungal 1,3(4)-beta-D-glucanase	3.2
GH16	EfM3.080050_1	Glycoside hydrolase family 16	3.7
GH16	EfM3.079050_1	Transglycosidase; links chitin to glucan	−2.7
GH17	EfM3.044280_1	GPI-anchored cell wall beta-1,3-endoglucanase	2.3
GH18	EfM3.024310_1	*Efe*-ChiA, Chitinase	−3.9
GH32	EfM3.074710_1	Beta-fructofuranosidase	3.2
GH5/GH15	EfM3.013890_1	*Efe*-GcnA, Beta-1,6-glucanase	−5.4
GH54/CBM42	EfM3.015180_1	Alpha-L-arabinofuranosidase	5.6
GH89	EfM3.029480_1	Alpha-N-acetylglucosaminidase	3.8
GT2	EfM3.049120_1	Chitin synthase 1	2.8
GT2	EfM3.080040_1	Glycosyltransferase family 2	5.0
GT25	EfM3.002010_1	LPS glycosyltransferase	−2.7
GT48	EfM3.026320_2	Beta 1,3 glucan synthase	7.2
GT90	EfM3.037010_1	Glycosyltransferase	−3.9
GT90	EfM3.041930_1	Glycosyltransferase family 90	−2.3
CBM50	EfM3.029340_1	LysM domain-containing	−5.1

^a^ A positive Log_2_ fold change (FC) value indicates that the gene was more highly expressed in the choke stroma tissue, and a negative fold change value indicates that the gene was more highly expressed in the asymptomatic inflorescence tissue.

**Table 7:** Expression of genes for glucosylceramide biosynthesis.

Gene (Gene Model)	Mean RPKM ^a^	Log_2_ FC ^b^	FDR *p*	Reference (Accession Number)
Serine palmitoyltransferase				
Subunit 1 (EfM3.026330)	73	NS	NS	[73] (AAK40364)
Subunit 2 (EfM3.013700)	604	2.6	0.0023	[73] (AAP47107)
3-Ketodihydrosphingosine reductase				
(EfM3.003080)	31	NS	NS	[74] (KMK61006)
Ceramide synthase				
(EfM3.037330)	134	4.9	0.0003	[75] (CBF77743)
Sphingolipid Δ4-desaturase				
(EfM3.079270)	1000	NS	NS	[76] (O59715)
Sphingolipid Δ8-desaturase				
(EfM3.018540)	708	2.6	0.0027	[77] (CBF77189)
Sphingolipid C9-methyltransferase				
(EfM3.016950)	829	4.2	9E-05	[77] (CBF81402)
Glucosylceramide synthase				
(EfM3.023750)	30	NS	NS	[77] (CBF77985)

^a^ RPKM value (reads per kilobase of exon model per million mapped reads) is the mean of the three choke stroma sequence replicates. ^b^ Fold change (FC) is the ratio of the largest mean RPKM values to the lowest mean RPKM values in the comparison of choke stroma tissues with asymptomatic inflorescence tissues. A positive fold change value indicates that the gene was more highly expressed in the choke stroma tissues. NS indicates that there was no statistical difference in gene expression between the asymptomatic inflorescence and stroma tissues at the false discovery rate (FDR)-adjusted *p*-value < 0.01. The statistical model used by the analysis program corrects for differences in library size, so the fold changes cannot be determined by simple algebraic calculations of the mean RPKM values of the two tissue types.

**Table 8:** Most *Epichloë festucae* alkaloid biosynthetic genes were more highly expressed in the asymptomatic inflorescence tissue than in the choke stroma tissue.

Gene (Gene Model)	Mean RPKM ^a^	Log_2_ FC ^b^	FDR *p*-Value
Peramine biosynthesis			
*perA*-2 (EfM3.018710)	24.7	−3.8	8.6 × 10^−10^
Ergot alkaloid biosynthesis			
*dmaW* (EfM3.065770)	2.2	−7.1	0.0006
*easF* (EfM3.049640)	3.2	−6.5	0.001
*easE* (EfM3.049630)	9.9	−12.0	1.38 × 10^−5^
*easC* (EfM3.065755)	1.5	NS	NS
*easD* (EfM3.065750)	<0.01	NS	NS
*easA* (EfM3.049660)	3.2	−7.9	0.0007
*easG* (EfM3.049650)	11.0	−14.0	1.44 × 10^−13^
*cloA* (EfM3.065760)	5.0	NS	NS
*lpsB* (EfM3.049620)	9.7	−10.8	8.37 × 10^−12^
*lpsA* (EfM3.063200)	1.3	NS	NS
*easH* (EfM3.049670)	17.6	NS	NS

^a^ RPKM value (reads per kilobase of exon model per million mapped reads) is the mean of the three asymptomatic inflorescence sequence replicates. ^b^ Fold change (FC) is the ratio of the largest mean RPKM values to the lowest mean RPKM values in the comparison of choke stroma tissues with asymptomatic inflorescence tissues. A negative fold change value indicates the gene was more highly expressed in the asymptomatic inflorescence tissue. NS indicates that there was no statistical difference in gene expression between the asymptomatic inflorescence and stroma tissues at the false discovery rate (FDR)-adjusted *p*-value < 0.01. The statistical model used by the analysis program corrects for differences in library size, so the fold changes cannot be determined by simple algebraic calculations of the mean RPKM values of the two tissue types.

**Table 9:** Differential gene expression of some *Epichloë festucae* non-ribosomal peptide synthetases and polyketide synthases between asymptomatic inflorescence tissue and choke stroma tissue at false discovery rate (FDR)-adjusted *p* < 0.01.

Gene Model	Mean RPKM ^a^	Log_2_ FC ^b^	FDR *p*-Value
Non-ribosomal peptide synthetases			
EfM3.059650	13	−6.5	1.2 × 10^−7^
EfM3.059660	12	−6.4	4.0 × 10^−10^
EfM3.081180	174	−8.0	0
EfM3.081210	223	−5.5	0
EfM3.059650	13	−6.5	1.2 × 10^−7^
Polyketide synthases			
EfM3.014820	6	−4.5	0.0017
EfM3.081190	108	−6.7	0

^a^ RPKM value (reads per kilobase of exon model per million mapped reads) is the mean of the three asymptomatic inflorescence sequence replicates. ^b^ Fold change (FC) is the ratio of the largest mean RPKM values to the lowest mean RPKM values in the comparison of choke stroma tissues with asymptomatic inflorescence tissues. A negative fold change value indicates that the gene was more highly expressed in the asymptomatic inflorescence tissue. The statistical model used by the analysis program corrects for differences in library size, so the fold changes cannot be determined by simple algebraic calculations of the mean RPKM values of the two tissue types.

**Table 10:** Over-represented plant gene ontology (GO) biological process categories of differentially expressed (DE) plant transcripts in choke stroma tissue (CS) and asymptomatic inflorescence (AI) using Fisher’s Exact Text at false discovery rate (FDR) <0.05 in Blast2GO. Only the most specific GO categories are presented.

DE	Biological Process GO Name	FDR	Nr DE	Nr Reference ^a^
CS > AI	Carbohydrate metabolic process	2.71 × 10^−6^	130	1804
CS > AI	Response to water deprivation	0.001	6	7
CS > AI	Transport	0.001	238	4328
CS > AI	RNA phosphodiester bond hydrolysis	0.004	19	146
CS > AI	Cell–cell signaling	0.008	3	0
CS > AI	Response to biotic stimulus	0.013	46	603
CS > AI	Lignin catabolic process	0.023	3	1
CS > AI	Cofactor catabolic process	0.048	4	7
CS > AI	Response to abscisic acid	0.048	4	7
CS < AI	Cell cycle	5.07 × 10^−23^	169	892
CS < AI	Flower development	4.58 × 10^−7^	58	317
CS < AI	Anatomical structure morphogenesis	8.17 × 10^−7^	70	442
CS < AI	Cellular component organization	1.00 × 10^−6^	382	4048
CS < AI	Cell differentiation	2.70 × 10^−4^	49	323
CS < AI	Lipid metabolic process	5.88 × 10^−4^	145	1397
CS < AI	Carbohydrate metabolic process	0.005	169	1765
CS < AI	Negative regulation of translation	0.029	6	10

^a^ Number of contigs in the plant assembly with the GO category annotation minus the number up- or down-regulated.

**Table 11:** Up-regulated plant genes in the choke stroma tissues in the gene ontology (GO) categories of response to biotic stimulus and response to water deprivation (false discovery rate <0.05).

Contig	Protein Annotation	Log_2_ FC ^a^
Response to biotic stimulus; GO:0009607		
TRINITY_DN38410_c0_g1_i1	1-aminocyclopropane-1-carboxylate oxidase 1	3.4
_contig_31681	Bet v 1 allergen	3.4
_contig_10385	Calcium-dependent protein kinase 12	2.2
TRINITY_DN34300_c1_g1_i1	Calcium-dependent protein kinase 12	2.3
_contig_24170	Class I chitinase	5.9
_contig_3002	E3 ubiquitin-protein ligase RGLG4	2.5
_contig_33432	Ethylene-responsive transcription factor ABR1-like	3.4
TRINITY_DN8188_c0_g1_i1	Histidine kinase 3	2.2
_contig_10489	L-type lectin-domain containing receptor kinase IV.1	3.2
_contig_12768	L-type lectin-domain containing receptor kinase IV.1	2.8
_contig_17875	L-type lectin-domain containing receptor kinase IV.1	2.4
_contig_2947	L-type lectin-domain containing receptor kinase IV.1	3.6
_contig_6101	L-type lectin-domain containing receptor kinase IV.1	2.0
TRINITY_DN14979_c0_g1_i1	L-type lectin-domain containing receptor kinase IV.1	3.8
_contig_10466	L-type lectin-domain containing receptor kinase IV.1-like	3.4
TRINITY_DN10040_c0_g1_i1	L-type lectin-domain containing receptor kinase IX.1-like	2.6
_contig_1059	Non-specific lipid-transfer protein	5.6
_contig_1420	Non-specific lipid-transfer protein	6.0
_contig_628	Non-specific lipid-transfer protein	6.4
TRINITY_DN15788_c0_g1_i1	Non-specific lipid-transfer protein	6.5
TRINITY_DN30775_c0_g1_i1	Non-specific lipid-transfer protein	6.5
TRINITY_DN40235_c0_g1_i1	Non-specific lipid-transfer protein	4.0
TRINITY_DN56021_c0_g1_i1	Non-specific lipid-transfer protein 3	6.6
TRINITY_DN34047_c0_g1_i1	Pathogenesis-related protein 10	3.2
TRINITY_DN34047_c0_g1_i3	Pathogenesis-related protein 10	2.4
_contig_13205	Pathogenesis-related protein 4	2.6
_contig_16686	Peroxidase 21-like	2.7
_contig_19669	Peroxidase 21-like	2.5
_contig_16216	Probable LRR receptor-like serine/threonine-protein kinase At1g74360	3.7
_contig_2758	Probable LRR receptor-like serine/threonine-protein kinase At1g74360	3.3
TRINITY_DN8117_c0_g1_i1	Probable RNA-dependent RNA polymerase 1	2.1
_contig_22174	Probable serine/threonine-protein kinase PBL25	2.4
_contig_1704	Protein NRT1/ PTR FAMILY 4.3-like (MFS superfamily protein)	2.9
_contig_18851	Protein synthesis inhibitor II	4.7
TRINITY_DN51159_c0_g1_i1	Putative eukaryotic translation initiation factor 4 gamma	2.7
_contig_1969	Putative lipid-transfer protein DIR1	4.5
_contig_2249	Putative lipid-transfer protein DIR1	5.9
_contig_4625	Putative lipid-transfer protein DIR1	6.3
_contig_5137	Putative lipid-transfer protein DIR1	5.4
_contig_544	Putative lipid-transfer protein DIR1	4.6
TRINITY_DN26665_c0_g1_i1	Putative ornithine aminotransferase	2.1
_contig_18027	Thaumatin-like protein	2.8
TRINITY_DN34173_c0_g1_i2	Thaumatin-like protein	2.9
_contig_26838	Triacylglycerol lipase 2	4.7
_contig_27	Triacylglycerol lipase 2	6.9
TRINITY_DN31985_c0_g1_i3	WRKY transcription factor	2.2
Response to water deprivation; GO:0009414		
_contig_691	LEA protein	3.1
TRINITY_DN26665_c0_g1_i1	Putative ornithine aminotransferase	2.1
TRINITY_DN31632_c1_g1_i1	Dehydrin DHN3	3.3
TRINITY_DN43898_c0_g1_i8	Heat shock protein 90-5, chloroplastic	2.1
TRINITY_DN63715_c0_g1_i1	Aquaporin PIP1-5	3.5
TRINITY_DN8188_c0_g1_i1	Histidine kinase 3	2.2

^a^ A positive Log_2_ fold change (FC) value indicates that the gene was more highly expressed in the choke stroma tissue.

## Supplementary Materials

The following are available online at https://www.mdpi.com/2076-2607/7/11/567/s1, Table S1: *Epichloë festucae* differentially expressed genes significantly down-regulated in the choke stroma tissue relative to the asymptomatic inflorescence tissue; Table S2: *Epichloë festucae* differentially expressed genes significantly more highly expressed in the choke stroma tissue than in the asymptomatic inflorescence tissue; Table S3: Expression of candidate host specialization genes; Table S4: Differentially expressed *Epichloë festucae* genes related to transport; Table S5: *Epichloë festucae* symbiosis-associated genes found not to be differentially expressed between the asymptomatic inflorescence tissue and the choke stroma tissue; Table S6: Overlap of *Epichloë festucae* differentially expressed genes from this study and the “core gene set”; Table S7: Differentially expressed plant genes.
