# Supplementary material for: Transcriptome Analysis of Choke Stroma and Asymptomatic Inflorescence Tissues Reveals Changes in Gene Expression in Both Epichloë festucae and Its Host Plant Festuca rubra subsp. rubra
Source: Microorganisms. 2019 Nov 16;7(11):567. doi: 10.3390/microorganisms7110567 (PMC6921078; doi:10.3390/microorganisms7110567)
Supplement: Supplementary file 1 [file microorganisms-07-00567-s001.zip › Table S3.docx]

**Supplementary Dataset S3.** Expression of candidate host specialization genes identified by Schirrmann et al. (2018)

| **Gene (gene model)** | **Mean RPKM^a^** | **Log_2_ FC^b^** | **FDR *p* value** |
| --- | --- | --- | --- |
| Pectin methylesterase (EfM3.008730) | 33.0 | -3.1 | 0.009 |
| Peroxidase 2 (EfM3.007770) | <0.01 | NS | NS |
| Endo-1,4-beta xylanase (EfM3.040190) | 145.3 | -3.4 | 2.0e-7 |
| Candidate effector (EfM3.008740) | 25.5 | NS | NS |

^a^ RPKM value (reads per kilobase of exon model per million mapped reads) is the mean of the three asymptomatic inflorescence sequence replicates.

^b^ Fold change (FC) is the ratio of the largest mean RPKM values to the lowest mean RPKM values in the comparison of choke stroma tissues with asymptomatic inflorescence tissues. Negative fold change value indicates the gene was more highly expressed in the asymptomatic inflorescence tissue. NS indicates there was no statistical difference in gene expression between the asymptomatic inflorescence and stroma tissues at the false discovery rate (FDR) adjusted *p* value < 0.01. The statistical model used by the analysis program corrects for differences in library size so the fold changes cannot be determined by simple algebraic calculations of the mean RPKM values of the two tissue types.
