# Supplementary material for: Transcriptome Analysis of Choke Stroma and Asymptomatic Inflorescence Tissues Reveals Changes in Gene Expression in Both Epichloë festucae and Its Host Plant Festuca rubra subsp. rubra
Source: Microorganisms. 2019 Nov 16;7(11):567. doi: 10.3390/microorganisms7110567 (PMC6921078; doi:10.3390/microorganisms7110567)
Supplement: Supplementary file 1 [file microorganisms-07-00567-s001.zip › Table S4.docx]

**Supplementary Dataset S4.** Differentially expressed *Epichloë festucae* genes related to transport at false discovery rate adjusted *p* < 0.01

| **Gene** | **Log_2_ FC^a^** |
| --- | --- |
| EfM3.017590_2 Major facilitator superfamily, possible iron siderophore transporter | 12.9 |
| EfM3.019610_1 *Efe*-MfsB; membrane transporter protein | 4.5 |
| EfM3.028310_1 Major facilitator superfamily, sugar transporter | 4.1 |
| EfM3.054480_1 ABC type transporter | 2.5 |
| EfM3.054490_2 Major facilitator superfamily, sugar transporter | 4.6 |
| EfM3.059060_1 Golgi transport complex subunit COG4 | 13.5 |
| EfM3.073030_1 Mitochondrial amino acid transporter arg-13 | 2.5 |
| EfM3.076540_1 Oligopeptide transporter OPT superfamily | 3.6 |
| EfM3.056950_1 Amino acid permease | 13.6 |
| EfM3.014910_1 Major facilitator superfamily, multidrug resistance transporter | 4.4 |
| EfM3.001040_2 Vacuolar protein sorting vps16 | 14.6 |
| EfM3.025350_1 Aquaglyceroporin | 3.7 |
| EfM3.038260_1 Magnesium-translocating P-type ATPase | 3.3 |
| EfM3.070700_1 Vacuolar protein sorting protein DigA | 12.7 |
| EfM3.073880_1 Aminophospholipid translocase | 3.9 |
| EfM3.014790_1 ABC multidrug transporter | -6.8 |
| EfM3.017940_1 Major facilitator superfamily transporter | -5.5 |
| EfM3.027570_1 Major facilitator superfamily, possible peptide transporter | -3.8 |
| EfM3.029870_1 Major facilitator superfamily transporter | -2.6 |
| EfM3.047170_1 Permease, cytosine/purine, uracil, thiamine, allantoin | -2.5 |
| EfM3.056220_1 ABC-type multidrug transport system | -5.7 |
| EfM3.069880_1 Major facilitator superfamily transporter | -4.0 |
| EfM3.082040_1 Major facilitator superfamily transporter | -2.4 |

^a^ Positive Log_2_ fold change (FC) value indicates the gene was more highly expressed in the choke stroma tissue and negative fold change value indicates the gene was more highly expressed in the asymptomatic inflorescence tissue.
