# Supplementary material for: Transcriptome Analysis of Choke Stroma and Asymptomatic Inflorescence Tissues Reveals Changes in Gene Expression in Both Epichloë festucae and Its Host Plant Festuca rubra subsp. rubra
Source: Microorganisms. 2019 Nov 16;7(11):567. doi: 10.3390/microorganisms7110567 (PMC6921078; doi:10.3390/microorganisms7110567)
Supplement: Supplementary file 1 [file microorganisms-07-00567-s001.zip › Table S5.docx]

**Supplementary Dataset S5.** *Epichloë festucae* genes known to be important for a normal symbiotic association with the host grass were not significantly differentially expressed in the choke stroma tissue relative to the asymptomatic inflorescence tissue.

| **Gene** | **Gene model** | **Mean RPKM^a^** | **Reference** |
| --- | --- | --- | --- |
| acy*A* | EfM3.048730 | 5 | Voisey et al. 2016^b^ |
| *bemA* | EfM3.080850 | 46 | Takemoto et al. 2011^c^ |
| *cclA* | EfM3.021140 | 8 | Lukito et al. 2019^d^ |
| *cdc*42 | EfM3.070900 | 46 | Kayano et al. 2018^e^ |
| *clrD* | EfM3.062280 | 49 | Chujo and Scott 2014^f^ |
| *cnaA*1 | EfM3.035130 | 76 | Mitic et al. 2018^g^ |
| *cnaA*2 | EfM3.051970 | 25 | Mitic et al. 2018 |
| *ezhB* | EfM3.069800 | 26 | Chujo and Scott 2014 |
| *hepA* | EfM3.043690 | 110 | Chujo et al. 2019^h^ |
| *kdmB* | EfM3.035320 | 18 | Lukito et al. 2019 |
| *laeA* | EfM3.069170 | 32 | Rahnama et al. 2019^i^ |
| *mkkA* | EfM3.016595 | 64 | Becker et al. 2015^j^ |
| *mobC* | EfM3.028150 | 27 | Green et al. 2016^k^ |
| *mpkA* | EfM3.074990 | 208 | Becker et al. 2015 |
| *noxA* | EfM3.065460 | 58 | Tanaka et al. 2006^l^ |
| *noxR* | EfM3.013400 | 81 | Takemoto et al. 2006^m^ |
| *pacC* | EfM3.009480 | 36 | Lukito et al. 2015^n^ |
| *plsA* | EfM3.019170 | 294 | Green et al. 2019^o^ |
| *proA* | EfM3.060970 | 149 | Tanaka et al. 2013^p^ |
| *racA* | EfM3.048590 | 162 | Tanaka et al. 2008^q^ |
| *rhgA* | EfM3.030930 | 1521 | Bassett et al. 2016^r^ |
| *sakA* | EfM3.073930 | 128 | Eaton et al. 2010^s^ |
| *sidN* | EfM3.029790 | 6 | Johnson et al. 2013^t^ |
| *so* (soft) | EfM3.015580 | 37 | Charlton et al. 2012^u^ |
| *symB* | EfM3.029010 | 163 | Green et al. 2017^v^ |
| *symC* | EfM3.029020 | 4 | Green et al. 2017 |
| *velA* | EfM3.049680 | 129 | Rahnama et al. 2018^w^ |

^a^ RPKM value (reads per kilobase of exon model per million mapped reads) is the mean of the three choke stroma sequence replicates. The expression of these genes in the asymptomatic inflorescence tissues was not significantly different from those of the choke stroma tissues.

^b^ Voisey, C.R., Christensen, M.T., Johnson, L.J., Forester, N.T., Gagic, M., Bryan, G.T., Simpson, W.R., Fleetwood, D.J., Card, S.D., Koolaard, J.P., Maclean, P.H., and Johnson, R.D. 2016. cAMP signaling regulates synchronised growth of symbiotic *Epichloë* fungi with the host grass *Lolium perenne*. Front. Plant Sci. 7:1546.

^c^ Takemoto, D., Kamakura, S., Saikia, S., Becker, Y., Wrenn, R., Tanaka, A., Sumimoto, H., and Scott, B. 2011. Polarity proteins Bem1 and Cdc24 are components of the filamentous fungal NADPH oxidase complex. Proc. Natl. Acad. Sci. U.S.A. 108:2861-2866.

^d^ Lukito, Y., Chujo, T., Hale, T.K., Mace, W., Johnson, L.J., and Scott, B. 2019. Regulation of subtelomeric fungal secondary metabolite genes by H3K4me3 regulators CclA and KdmB. Mol. Microbiol. doi:10.1111/mmi.14320

^e^ Kayano, Y., Tanaka, A., and Takemoto, D. 2018. Two closely related Rho GTPases, Cdc42 and RacA, of the endophytic fungus *Epichloë festucae* have contrasting roles for ROS production and symbiotic infection synchronized with the host plant. PLoS Pathog. 14:e1006840.

^f^ Chujo, T., and Scott, B. 2014. Histone H3K9 and H3K27 methylation regulates fungal alkaloid biosynthesis in a fungal endophyte–plant symbiosis. Mol. Microbiol. 92:413-434.

^g^ Mitic, M., Berry, D., Brasell, E., Green, K., Young, C.A., Saikia, S., Rakonjac, J., and Scott, B. 2018. Disruption of calcineurin catalytic subunit (*cnaA*) in *Epichloë festucae* induces symbiotic defects and intrahyphal hyphae formation. Mol. Plant Pathol. 19:1414-1426.

^h^ Chujo, T., Lukito, Y., Eaton, C.J., Dupont, P.-Y., Johnson, L.J., Winter, D., Cox, M.P., and Scott, B. 2019. Complex epigenetic regulation of alkaloid biosynthesis and host interaction by heterochromatin protein I in a fungal endophyte-plant symbiosis. Fungal Genet. Biol. 125:71-83.

^i^ Rahnama, M., Maclean, P., Fleetwood, D.J., and Johnson, R.D. 2019. The LaeA orthologue in *Epichloë festucae* is required for symbiotic interaction with *Lolium perenne*. Fungal Genet. Biol. 129:74-85.

^j^ Becker, Y., Eaton, C.J., Brasell, E., May, K.J., Becker, M., Hassing, B., Cartwright, G.M., Reinhold, L., and Scott, B. 2015. The fungal cell-wall integrity MAPK cascade is crucial for hyphal network formation and maintenance of restrictive growth of *Epichloë festucae* in symbiosis with *Lolium perenne*. Mol. Plant-Microbe Interact. 28:69-85.

^k^ Green, K.A., Becker, Y., Fitzsimons, H.L., and Scott, B. 2016. An *Epichloë festucae* homologue of MOB3, a component of the STRIPAK complex, is required for the establishment of a mutualistic symbiotic interaction with *Lolium perenne*. Mol. Plant Pathol. 17:1480-1492.

^l^ Tanaka, A., Christensen, M.J., Takemoto, D., Park, P., and Scott, B. 2006. Reactive oxygen species play a role in regulating a fungus–perennial ryegrass mutualistic interaction. Plant Cell 18:1052-1066.

^m^ Takemoto, D., Tanaka, A., and Scott, B. 2006. A p67^Phox^-like regulator is recruited to control hyphal branching in a fungal–grass mutualistic symbiosis. Plant Cell 18:2807-2821.

^n^ Lukito, Y., Chujo, T., and Scott, B. 2015. Molecular and cellular analysis of the pH response transcription factor PacC in the fungal symbiont *Epichloë festucae*. Fungal Genetics and Biology 85:25-37.

^o^ Green, K.A., Eaton, C.J., Savoian, M.S., and Scott, B. 2019. A homologue of the fungal tetraspanin Pls1 is required for *Epichloë festucae* expressorium formation and establishment of a mutualistic interaction with *Lolium perenne*. Mol. Plant Pathol. doi:10.1111/mpp.12805.

^p^ Tanaka, A., Cartwright, G.M., Saikia, S., Kayano, Y., Takemoto, D., Kato, M., Tsuge, T., and Scott, B. 2013. ProA, a transcriptional regulator of fungal fruiting body development, regulates leaf hyphal network development in the *Epichloë* *festucae*–*Lolium perenne* symbiosis. Mol. Microbiol. 90:551-568.

^q^ Tanaka, A., Takemoto, D., Hyon, G.-S., Park, P., and Scott, B. 2008. NoxA activation by the small GTPase RacA is required to maintain a mutualistic symbiotic association between *Epichloë festucae* and perennial ryegrass. Mol. Microbiol. 68:1165-1178.

^r^ Bassett, S.A., Johnson, R.D., Simpson, W.R., Laugraud, A., Jordan, T.W., and Bryan, G.T. 2016. Identification of a gene involved in the regulation of hyphal growth of *Epichloë festucae* during symbiosis. FEMS Microbiol. Lett. 363, doi: 10.1093/femsle/fnw214.

^s^ Eaton, C.J., Cox, M.P., Ambrose, B., Becker, M., Hesse, U., Schardl, C.L., and Scott, B. 2010. Disruption of signaling in a fungal-grass symbiosis leads to pathogenesis. Plant Physiol. 153:1780-1794.

^t^ Johnson, L.J., Koulman, A., Christensen, M., Lane, G.A., Fraser, K., Forester, N., Johnson, R.D., Bryan, G.T., and Rasmussen, S. 2013. An extracellular siderophore is required to maintain the mutualistic interaction of *Epichloë festucae* with *Lolium perenne*. PLOS Pathog. 9:e1003332.

^u^ Charlton, N.D., Shoji, J.-Y., Ghimire, S.R., Nakashima, J., and Craven, K.D. 2012. Deletion of the fungal gene *soft* disrupts mutualistic symbiosis between the grass endophyte *Epichloë festucae* and the host plant. Eukaryot. Cell 11:1463-1471.

^v^ Green, K.A., Becker, Y., Tanaka, A., Takemoto, D., Fitzsimons, H.L., Seiler, S., Lalucque, H., Silar, P., and Scott, B. 2017. SymB and SymC, two membrane associated proteins, are required for *Epichloë festucae* hyphal cell–cell fusion and maintenance of a mutualistic interaction with *Lolium perenne*. Mol. Microbiol. 103:657-677.

^w^ Rahnama, M., Johnson, R.D., Voisey, C.R., Simpson, W.R., and Fleetwood, D.J. 2018. The global regulatory protein VelA is required for symbiosis between the endophytic fungus *Epichloë festucae* and *Lolium perenne*. Mol. Plant-Microbe Interact. 31:591-604.
