# Supplementary material for: Transcriptome Analysis of Choke Stroma and Asymptomatic Inflorescence Tissues Reveals Changes in Gene Expression in Both Epichloë festucae and Its Host Plant Festuca rubra subsp. rubra
Source: Microorganisms. 2019 Nov 16;7(11):567. doi: 10.3390/microorganisms7110567 (PMC6921078; doi:10.3390/microorganisms7110567)
Supplement: Supplementary file 1 [file microorganisms-07-00567-s001.zip › Table S6.docx]

**Supplementary Dataset S6.** Overlap of *Epichloë festucae* differentially expressed genes with the “core gene set” from Eaton et al. (2015).

|  | **Tissue of higher expression** | |
| --- | --- | --- |
| **Gene model and protein annotation** | **Inflorescence or stroma^a^** | **Wild type or mutant interaction^b^** |
| EfM3.001580 Uncharacterized protein | Stroma | Mutant |
| EfM3.005300 *Efe*-PrtC, subtilisin-like protease, secreted | Inflorescence | Wild type |
| EfM3.013550 NAD+ dependent glutamate dehydrogenase | Stroma | Mutant |
| EfM3.016770 *Efe*-SspM, candidate effector protein | Inflorescence | Wild type |
| EfM3.018170 Uncharacterized, NC12 | Inflorescence | Wild type |
| EfM3.027570 Major facilitator superfamily domain | Inflorescence | Mutant |
| EfM3.028490 Uncharacterized | Inflorescence | Wild type |
| EfM3.040190 Endo-1,4-beta-xylanase, secreted | Inflorescence | Mutant |
| EfM3.041770 Candidate effector protein | Inflorescence | Mutant |
| EfM3.044610 Uncharacterized protein, small secreted | Inflorescence | Wild type |
| EfM3.044630 Candidate effector protein | Inflorescence | Mutant |
| EfM3.045520 Uncharacterized, small secreted | Inflorescence | Wild type |
| EfM3.047600 Uncharacterized protein | Stroma | Wild type |
| EfM3.048860 Uncharacterized | Inflorescence | Mutant |
| EfM3.051900 Uncharacterized | Inflorescence | Mutant |
| EfM3.056220 ABC-type multidrug transport system | Inflorescence | Wild type |
| EfM3.056300 Ubiquitin/ribosomal protein S27a fusion | Inflorescence | Wild type |
| EfM3.057830 Cholinesterase | Inflorescence | Mutant |
| EfM3.064250 Uncharacterized protein | Stroma | Mutant |
| EfM3.069610 Uncharacterized protein | Inflorescence | Mutant |
| EfM3.073830 Uncharacterized protein | Stroma | Mutant |
| EfM3.074710 Secreted invertase | Stroma | Mutant |

^a^ Statistically different at false discovery rate adjusted *p* < 0.01 and Log_2_ fold change > 2.

^b^ Eaton, C.J., Dupont, P.-Y., Solomon, P., Clayton, W., Scott, B., and Cox, M.P. 2015. A core gene set describes the molecular basis of mutualism and antagonism in *Epichloë* spp. Mol. Plant-Microbe Interact. 28:218-231.
